# Time Until Proof of Credentials Significantly Decreases With the Use of Blockchain Technology and the Document Management System

**DOI:** 10.7759/cureus.48920

**Published:** 2023-11-16

**Authors:** Elizabeth A Tissier, Anapaula Berglund, Gabrielle J Johnson, Zakary A Sanzone, Anna P Goodbread, Heath Parker, John Lucas, David Kashmer

**Affiliations:** 1 Simulation Center, Edward Via College of Osteopathic Medicine, Auburn, USA; 2 Medical Education and Simulation, Edward Via College of Osteopathic Medicine, Tuscaloosa, USA; 3 Administration, Edward Via College of Osteopathic Medicine, Auburn, USA; 4 Simulation Center, Edward Via College of Osteopathic Medicine, Blacksburg, USA

**Keywords:** educational certificate verification, user authentication, verification, document management, credentialing, nft, blockchain

## Abstract

Background and objective

Physician credentialing and verification in the medical education setting are challenging for the modern workforce. The credentials verification process may be time-consuming and challenging for participants. Blockchain technology is a potential resource for authenticating records with reduced administrative burden and time spent. This study investigates whether the use of blockchain technology reduces the time until verification of a participant’s credentials.

Methods

An anonymous letter designation was assigned to 23 medical students. All students enrolled in, and completed, a course designed and run by the Edward Via College of Osteopathic Medicine at Auburn (VCOM) as part of the routine medical education curriculum. At the completion of the training, a credentials certificate was produced, which showed course completion. The anonymous letter designation was utilized in the creation of the certificates. The letter designations were shared with an anonymous investigator. No student names were shared with the investigator. The investigator posed as an employing/credentialing entity and contacted VCOM to record the time required to verify the credentials certificate indicating course completion. The elapsed time until credentials verification was completed for each student in the current system (CS) was recorded. Subsequently, the credentials certificate was minted as a blockchain-based, non-fungible token (NFT) and uploaded to a document software management system. An investigator again posed as an employing/credentialing entity and utilized this system to verify the credentials of the 23 students in the study using the NFT system. The times elapsed until verification of credentials were recorded as the NFT pathway. Data from the NFT pathway and non-NFT pathway were compiled and reviewed.

Results

Data were normally distributed per the Andersen-Darling Test. A t-test (Welch’s method) was performed. The mean time of 111,214 seconds (30.89 hours or 1.29 days) in the CS varied significantly from the mean time of 14 seconds in the NFT blockchain system (p<0.01). The standard deviation of 56,568 seconds in CS varied significantly from 9.9178 seconds in the NFT blockchain (p<0.01).

Conclusions

The NFT/blockchain system reduces the mean time until the credential verification is completed and reduces the variance seen in time until credentialing is completed. The NFT/blockchain system may significantly bring down the administrative burden and time spent in the credentialing process.

## Introduction

The process of credentialing physicians in medical staff offices by using current systems is time-consuming and inefficient. The need to maintain credentialing authenticity is of the utmost importance; however, the systems currently in place have a difficult time combating academic fraud and substantiating credentials. For example, it has been estimated that in the United States, there are currently two million fake degree certificates in circulation and 300 unauthorized universities operating [1]. These same ineffective processes are also used for medical student credentialing and for demonstrating course completion to external sources. There are similar drawbacks to these systems when it comes to efficiency and credential accuracy. This study investigates whether the use of novel blockchain and non-fungible token (NFT) technology helps reduce the time until the demonstration of credentials while maintaining credential integrity.

Blockchain frameworks have been incorporated into many industries in recent years. Studies have been performed to determine blockchain’s viability as a network for “patient-centered access” as well as a system for health information exchange [2]. As these systems have shown promise in previous studies, we hope that blockchain technology can also help improve current systems for medical school credentialing. It has already been shown that blockchain can significantly improve security in terms of student credentials by linking certificates to user identity via biometrics, identification numbers, and personal data [3]. However, no technology is perfect and every technology has drawbacks. Previous studies have highlighted the potential downsides of blockchain technology including a larger carbon footprint and potential mistrust associated with the broader use of the technology. Since blockchain is a decentralized network, it should promote transparency; however, the technology is currently not well-regulated, which could cause concerns regarding inappropriate access and overall security of the system [4]. 

As we enter into a more technology-focused future, benefits, as well as possible downsides of new technology, should be weighed to determine the effectiveness of a new process. Even though certain regulations may be lacking, essential characteristics have been defined in order to have a well-secure blockchain framework in place. These include decentralization, transparency, anonymity, and a consensus-based, immutable, and open-sourced milieu [5]. This paper hopes to prove that the benefits of using NFTs and blockchain far outweigh the possible flaws within the system.

## Materials and methods

Twenty-three randomly selected, second-year medical students were enrolled in and completed a course designed and run by the Edward Via College of Osteopathic Medicine at Auburn (VCOM). At the completion of the training, a certificate was provided, demonstrating the completion and the degree of competency. Using the secret shopper technique, an investigator posing as an employing entity contacted VCOM requesting the verification of these credentials. The time until verification of these credentials was recorded using the current credentialing entity.

**Figure 1:**
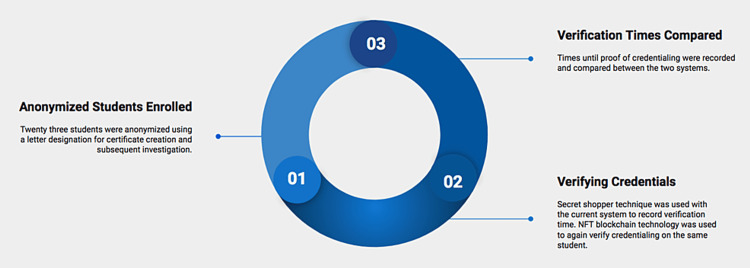
Credentialing workflow This figure depicts the workflow used in the new NFT credentialing system NFT: non-fungible token

Subsequently, the credentials certificate (with an anonymous letter designation and no student name) was minted as an NFT and uploaded to the blockchain software (ArchiveCore, Roanoke, VA). An investigator utilized this system to verify the credentials of the randomly selected 23 students when contacted by the anonymous employing entity. The time until proof of credentialing was recorded using the NFT pathway. Figure 1 illustrates the working steps using both systems to compare the verification times for data analysis. Student anonymity was maintained throughout this process by creating a key linking a letter designation to student names. Only the letter designation was shared with the investigator. The letter designation was utilized for certificate creation, NFT upload, and subsequent queries.

Data from the NFT pathway and non-NFT pathway was compiled and reviewed using MiniTab (version 21.2). Data analysis was performed using the two-sample t-test, two-sample standard deviation test, and test of variance. These tests provided an adequate comparison between the two systems in order for a significant difference to be established.

## Results

**Figure 2:**
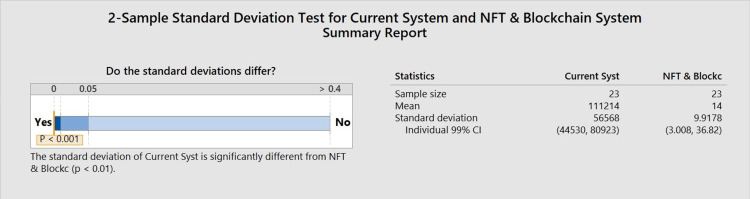
Standard deviation test This graph depicts the difference in standard deviations between the current system and the NFT blockchain system. The orange line indicates the significance level (p<0.0001) when the standard deviation of the current system was compared to the standard deviation of the NFT blockchain system. The significance level being <0.0001 designates this data as statistically significant NFT: non-fungible token

**Figure 3:**
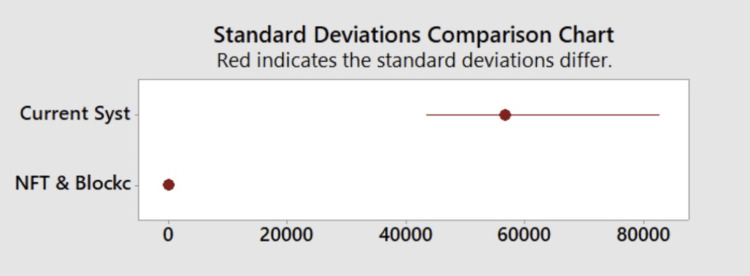
Standard deviation comparison chart This figure shows the comparison of the standard deviation of the current system with the standard deviation of the NFT blockchain system. The current system is listed on top with the red dot indicating the mean time for verification of credentials. The red horizontal line indicates the standard deviation of the data for the current system. The bottom red dot indicates the mean time for verification of credentials, and, due to scaling, the standard deviation for the NFT blockchain system could not be shown. The scale is from zero to 80,000 seconds to accommodate large differences in standard deviations between the two methods while still depicting both systems on the same figure NFT: non-fungible token

Significant differences were noted in verification time between the current system and the NFT/blockchain system. As shown in Figure 2, the standard deviation of the current system was 56,568 seconds, which varied significantly (p<0.001) from the standard deviation of the NFT/blockchain system (9.9178) seconds. Figure 3 shows the standard deviation of the current system compared to the standard deviation of the NFT/blockchain system. The NFT/blockchain system had a significantly lower standard deviation, showing the consistency of the NFT/blockchain system and how it can provide a dependable service when it comes to accessing credentials.

**Figure 4:**
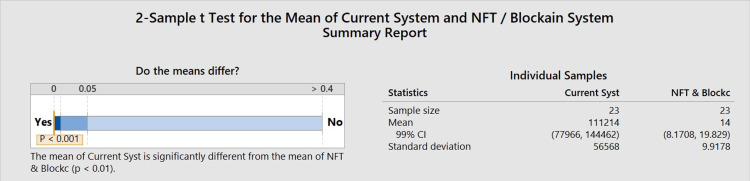
Sample t-test This graph compares the mean time for verification for the current system and the NFT blockchain verification system. The mean time for verification for the current system was 111,214 seconds. The mean time for verification for the NFT blockchain system was 14 seconds. This difference was a statistically significant difference with a p-value of <0.001. The orange line indicates a statistically significant p-value <0.001 NFT: non-fungible token

**Figure 5:**
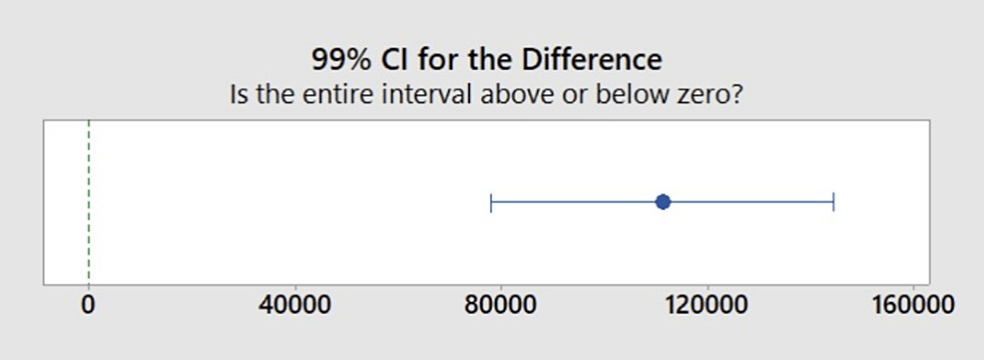
Confidence interval The confidence interval quantifies the uncertainty associated with estimating the difference in means between the current system and the NFT blockchain system. The data indicates that investigators can be 99% confident that the true difference is between 77,952 and 144,448. The mean time for verification for the current system was 111,214 seconds. The mean time for verification for the NFT blockchain system was 14 seconds NFT: non-fungible token

Figure 4 shows a comparison of the mean time to verification between the current system and the NFT/blockchain system. The current system took 111,214 seconds (30.89 hours, 1.29 days), which varied significantly (p<0.01) from the NFT/blockchain system, averaging 14 seconds for credential verification. Figure 5 shows the confidence interval for the difference between the mean wait times for credential verification for the current system and the NFT/blockchain system. Based on this data, it can be concluded that the NFT/blockchain system was significantly faster at accessing credentials.

By standardizing the credentialing process, there was not only a decrease in the time required for access to credentials but also a decrease in the variation between wait times. This shows that the system is faster and more consistent than the system currently in place, with the added benefit of reduced administrative burden. The results show the efficiency achieved by using NFT/blockchain for credential verifications.

## Discussion

Blockchain technology has been developed for many applications since it was introduced in 2008. While it is most popularly known for its association with the popular cryptocurrency Bitcoin, it has also been involved in government projects and other financial activities, as well as electronic healthcare records (EHR) for many years [6]. Implementing blockchain into EHR was a much-needed change, as the previous paper-based system had not been able to retrieve information properly and efficiently, especially in emergency settings. Information could also easily be lost and damaged, with the maintenance of these documents being quite time-consuming and requiring a lot of energy. Adding blockchain to the system enabled more data to be stored and secured with faster retrieval times. It has been shown to improve interoperability and access across organizations [7,8]. Even with the magnitude of information stored within the blockchain server, the longest wait time for information using the system was 14ms, exceedingly faster than current systems [9]. Several studies have demonstrated that the use of blockchain took less time than standard centralized storage schemes even with the additional overhead of user authentication [10]. The blockchain system also has the added benefit of allowing easier sharing between different departments within the hospital setting. It provides easier access to information for those within the system, where rigorous requirements were once needed to ensure security [11].

The applications for blockchain systems are constantly evolving with a new focus on its uses in higher education. An article from 2018 discussed the need for an easier and more secure way to immediately access credentials in higher education [12]. Blockchain solves this problem by providing an efficient way to access information that is reliable and tamper-proof, due to its better security. Blockchain works as a “decentralized” server that solves the problem of cybercrimes and falsification of academic records [13]. Research on this topic has extended to a system that would provide secure access on a global scale. A project was proposed in 2018, which involved using blockchain as a globally trusted credit system for higher education as a way to provide a more simplified and transparent system to overcome language and administrative barriers [14].

Credentialing of medical professionals is a time-consuming process for clinicians, training facilities, and healthcare employers. The current system used at this institution includes emailing or calling the institution for information and then waiting, on average, 1.3 days for a confirmation of the certification. The current system is very time-consuming and requires a substantial amount of administrative work. Technology has devised methods to reduce the amount of administrative burden in situations like this, especially in healthcare. ChatGPT, for example, can be used as a virtual assistant for doctors and nurses to aid in charting and medical record-keeping by automatically summarizing key details in patient records [15]. This reduces the time, energy, and resources needed to perform administrative tasks. The need for a more efficient and simplified system led to the addition of the blockchain system, using NFTs as student credits. Blockchain’s efficiency and precision have been demonstrated in other studies as well as in its many other applications such as medical supply chain, mobile health, biomedical databases, insurance claims, and EMRs [16]. With this new addition, we witnessed a drastic decrease in the time spent, along with a decrease in the administrative burden that is currently required to maintain credentialing at this institution. This paper, along with others, shows that the addition of the blockchain system makes a significant impact in terms of the efficiency and effectiveness of the current credentialing system.

Limitations

This study employed a relatively small volume of data to test the abilities of the blockchain system. Further investigations should be performed using larger data sets in order to assess the system's competency and speed with high data volumes. As the system becomes more saturated with data, it should be tested for signs of system overload and slower retrieval speeds.

## Conclusions

Blockchain technology has been used globally in different avenues including the healthcare field. In this article, the potential to implement blockchain technology in the credentialing of medical professionals was evaluated to assess its efficiency in terms of reducing time and administrative burden. The credentialing process without blockchain is a time-consuming process for all parties involved; however, by removing intermediaries from the process, blockchain can significantly enhance and revolutionize the process of credentialing. The main objective of this study was to determine whether using NFTs would reduce the time it takes to verify credentials. The results demonstrated that using the NFT/blockchain system significantly shortened the length of time required to verify participant’s credentials compared to the current system, which decreased the administrative burden. The NFT system also decreased the variation in times required to obtain credential verification compared to the current system.

The proposed NFT system addressed the time and administrative burden to bring about a more efficient and effective credentialing system. The data from this study supports previous findings from other papers regarding the blockchain system’s efficiency and consistency as well as its many uses in higher education. Blockchain's ability to provide modern solutions to professional credentialing shows that there is a need to revise current practices. Additional investigations into the topic of student access to these records may provide solutions for further system improvement and verification ease. Future plans for the NFT/blockchain system may include deploying the system more broadly to enhance not only administration but also students' access to verify certifications more easily. Any further plans for this project will have the main goal of providing easier access and reducing the challenges within the current system.
